# Diagnostic Accuracy of Synovial Calprotectin in Megaprosthetic Reconstructions: A Prospective Cohort Study from a Tertiary Sarcoma Center

**DOI:** 10.3390/cancers18101511

**Published:** 2026-05-08

**Authors:** Panayiotis Gavriil, Pavlos Altsitzioglou, Ioannis Trikoupis, Efthalia Maleka, Panagiotis Briassoulis, Jendrik Hardes, Panayiotis Papagelopoulos, Vasileios Kontogeorgakos

**Affiliations:** 1 First Department of Orthopaedics, ATTIKON University General Hospital, National and Kapodistrian University of Athens, 12461 Athens, Greece; gavriilpan@gmail.com (P.G.); pavlosaltsi@gmail.com (P.A.); itrikoupis@med.uoa.gr (I.T.); med11p1160099@med.uoc.gr (E.M.);; 2 Second Department of Anesthesiology, ATTIKON University General Hospital, National and Kapodistrian University of Athens, 12461 Athens, Greece; briaspan@med.uoa.gr; 3 Klinik für Tumororthopädie, Universitätsklinikum Essen, 45143 Essen, Germany; jendrik.hardes@uk-essen.de

**Keywords:** periprosthetic joint infection, calprotectin, megaprosthesis, diagnostic accuracy, musculoskeletal oncology, limb salvage

## Abstract

Background/Objectives: Diagnosing periprosthetic joint infection (PJI) after megaprosthetic reconstruction may be difficult due to altered inflammatory responses, extensive prior surgery, and the limited performance of conventional criteria such as the 2018 ICM score. Synovial calprotectin is a rapid neutrophil-derived biomarker that may improve diagnostic accuracy in this challenging setting. The primary aim of this study was to evaluate the diagnostic performance of synovial calprotectin in detecting periprosthetic infection in patients treated with tumor megaprostheses; secondary aims included comparison with ICM classification, assessment in infection classification-inconclusive cases, and exploratory performance in patients with low CRP. Methods: This prospective study included 20 consecutive megaprosthesis patients evaluated for suspected PJI at ATTIKON University Hospital, Athens, with a minimum follow-up of 1 year after biomarker testing. Synovial calprotectin was measured using a lateral-flow assay (positive ≥ 50 mg/L) and compared with a predefined infection reference standard. ICM final status (0 = aseptic, 1 = inconclusive, 2 = infected) was recorded for all cases. Other synovial biomarkers (α-defensin, leukocyte esterase, synovial D-dimer) were not routinely available. The cohort had a mean age of 52.9 ± 22.5 years, 70% were male, and reconstructions involved the knee (80%), hip (15%), and humerus (5%). Preoperative cultures were positive in 40%, the median systemic WBC was 7100/μL, and the median time from last surgery to testing was 1.0 years (IQR 0.46–2.0). Among infected cases, the most common microorganisms were coagulase-negative staphylococci (61.5%) and *Staphylococcus aureus* (23.1%), with 30.8% demonstrating polymicrobial infection. Results: Thirteen of 20 patients (65%) were classified as infected. Using the ≥50 mg/L threshold, synovial calprotectin demonstrated high apparent diagnostic accuracy in this exploratory cohort, and no false positives, yielding a sensitivity of 92.3%, specificity of 100%, PPV of 100%, NPV of 87.5%, LR+ = ∞, and LR− = 0.08. The AUC for continuous values was 1.00. Agreement with the ICM final classification was substantial (κ = 0.76), with no directional discordance (McNemar *p* = 1.00). Among the three ICM-inconclusive cases, calprotectin correctly reclassified two (66.7%). In patients with low CRP (<10 mg/L), a clinically difficult subgroup, calprotectin maintained strong performance (sensitivity 75%, specificity 100%, NPV 85.7%). Conclusion: Synovial calprotectin demonstrated promising diagnostic performance for PJI in megaprosthesis patients, with high sensitivity and specificity, and substantial agreement with the 2018 ICM criteria. It successfully clarified most ICM-inconclusive cases and remained reliable even in patients with low CRP. These findings support calprotectin as a valuable adjunctive biomarker in the complex diagnostic environment of megaprosthetic reconstruction and justify further validation in larger cohorts.

## Simple Summary

Periprosthetic infection after tumor resection and megaprosthetic reconstruction surgery is difficult to diagnose because standard blood and joint fluid tests often perform poorly in these complex reconstructions. In this prospective study from a tertiary sarcoma center, we evaluated whether synovial calprotectin, a rapid marker measured in joint fluid, can help identify periprosthetic joint infection in patients with megaprostheses. The study included 20 patients, of whom 13 were classified as infected and 7 as non-infected. In this cohort, calprotectin showed high apparent diagnostic accuracy, correctly identifying most infected cases and producing no false-positive results. It also remained useful when C-reactive protein levels were low and helped clarify cases that were inconclusive according to standard diagnostic criteria. These findings suggest that synovial calprotectin may be a practical adjunct for clinical decision-making in orthopedic oncology patients with suspected implant infection.

## 1. Introduction

Megaprosthetic reconstruction is widely used for limb salvage following oncological resection and extensive bone loss or revision arthroplasty, but it is associated with substantial complication rates, particularly periprosthetic joint infection (PJI). Infection rates commonly exceed 10–20%, with similar vulnerability reported in non-oncologic megaprosthesis patients and even higher rates in high-risk anatomical locations [1,2,3,4]. Management of PJI in megaprosthesis patients is especially challenging due to extensive soft-tissue compromise, the mechanical demands and size of these implants, and the frequent presence of significant medical comorbidities or prior oncologic treatments. These factors collectively impair local and systemic host defenses, leading to poorer outcomes and higher rates of treatment failure despite aggressive surgical intervention [1,5,6].

Accurate diagnosis of periprosthetic joint infection (PJI) is essential in megaprosthesis patients, both to ensure prompt identification and treatment of true infections and to avoid unnecessary revision surgeries, which in this population are high-risk and associated with poor outcomes and significant complication rates [7]. Traditional serum and synovial markers demonstrate limited diagnostic utility in megaprosthesis cases due to altered inflammatory responses, extensive soft-tissue compromise, prior oncologic treatments, and immunosuppression. As a result, CRP, ESR, synovial WBC, and PMN% frequently fail to differentiate infected from aseptic presentations [8,9]. Similarly, the 2018 International Consensus Meeting (ICM) criteria, although validated for routine hip and knee arthroplasty, often produce inconclusive classifications in megaprosthesis patients, further complicating surgical decision-making [10,11]. These challenges highlight the clinical need for a diagnostic tool with a high negative predictive value, capable of reliably excluding infection and preventing unnecessary high-morbidity revisions in this vulnerable patient group.

Early and accurate diagnostic information is essential in suspected megaprosthetic PJI because treatment success declines sharply with delays, and the choice between debridement, one-stage, or two-stage revision depends critically on the timing of symptom onset [12,13]. Rapid diagnostics are therefore valuable for guiding timely surgical planning, yet access to reliable point-of-care tools remains limited. Traditional rapid tests such as leukocyte esterase strips or serum inflammatory markers show inconsistent performance in detecting PJI and may be difficult to interpret in patients with compromised immune responses. Although newer assays, including synovial α-defensin, lateral-flow immunoassays, and laboratory-based synovial biomarkers, have demonstrated strong performance in conventional arthroplasty populations, their use is constrained by high cost, laboratory dependency, and limited availability in many institutions [14,15,16]. These limitations highlight the ongoing need for a rapid, practical, and broadly accessible biomarker capable of providing reliable early diagnostic information in patients with megaprostheses.

Calprotectin is a neutrophil-derived protein that becomes highly concentrated in infected synovial fluid, making it a biologically strong candidate marker for periprosthetic joint infection [17]. It can be quantified rapidly using lateral-flow assays that provide results within minutes and require minimal laboratory infrastructure. Multiple clinical studies and systematic reviews report high diagnostic accuracy for synovial calprotectin in conventional arthroplasty, with sensitivities and specificities frequently exceeding 90% [18,19,20]. Early validation of lateral-flow testing also demonstrated strong rule-out performance using a ≥50 mg/L threshold [21].

Despite this growing body of evidence, virtually all evaluations of synovial calprotectin have focused on standard hip and knee arthroplasty, leaving its performance in megaprosthesis patients largely unexplored.

The primary aim of this study was to evaluate the diagnostic accuracy of synovial calprotectin for detecting periprosthetic joint infection in patients who underwent reconstruction with megaprostheses. Secondary aims included assessing its performance in the subgroup of patients with low systemic inflammatory markers (CRP < 10 mg/L), examining its agreement with the 2018 ICM infection classification, and determining its potential to clarify cases categorized as inconclusive by the same classification system. A comparative analysis with non-megaprosthesis arthroplasty patients was additionally performed to contextualize calprotectin performance within the broader spectrum of reconstructive procedures.

This work represents the first prospective diagnostic accuracy study of synovial calprotectin in patients with megaprostheses. By focusing on a clinical setting in which diagnostic uncertainty is associated with substantial morbidity, this study provides evidence with important implications for surgical decision-making. If further validated, synovial calprotectin could become an integral component of the diagnostic pathway for megaprosthetic PJI, particularly as a rule-out test to help prevent unnecessary and high-risk revision procedures.

## 2. Materials and Methods

This was a prospective observational diagnostic accuracy study evaluating the performance of synovial calprotectin for identifying periprosthetic joint infection (PJI) in patients with tumor-related megaprosthetic reconstructions. Synovial fluid sampling and index test application were performed at the time of initial evaluation for suspected infection, and infection status was established using a predefined reference standard. In addition, a separate non-megaprosthesis arthroplasty cohort was assembled for exploratory matched comparative analyses.

The study was designed and reported in accordance with the STARD 2015 guidelines for diagnostic accuracy research [22].

Beginning in September 2023, all consecutive adult patients presenting to our tertiary referral sarcoma and arthroplasty center with clinical suspicion of periprosthetic joint infection (PJI) involving a previously implanted tumor-related megaprosthesis placed for primary malignant bone tumor or metastatic disease were prospectively enrolled. Synovial calprotectin testing was performed at the time of diagnostic aspiration, and all enrolled patients completed a minimum of one year of clinical follow-up after biomarker sampling to ensure accurate determination of infection status.

For exploratory matched comparative analyses, a separate pool of non-megaprosthesis arthroplasty patients evaluated during the same study period with the same diagnostic workflow and definitive infection reference classification was also identified.

Patients were included if they were ≥18 years old, had undergone prior arthroplasty of the index joint being evaluated for possible infection, and underwent diagnostic synovial fluid aspiration due to the presence of clinical signs, symptoms, or laboratory abnormalities suggestive of PJI. Additional inclusion criteria required adequate synovial aspirate volume for calprotectin testing and availability of complementary diagnostic data, including routine synovial analysis (cell count, differential, culture) and serum inflammatory markers (CRP, ESR). All included patients subsequently underwent revision surgery after biomarker sampling, during which intraoperative tissue cultures were collected as part of the diagnostic reference evaluation. This requirement ensured that all cases included in the primary analysis had operative and microbiological data available for reference standard adjudication.

Patients were excluded if they lacked signs, symptoms, or laboratory findings indicative of infection, if synovial aspiration was performed within the first three postoperative weeks, if synovial fluid was insufficient for testing, if a definitive infection reference standard could not be established, or if essential clinical or laboratory data relevant to diagnostic accuracy analysis were missing. No participants were excluded due to assay malfunction or processing failure.

The study was conducted at the First Department of Orthopaedics, National and Kapodistrian University of Athens, ATTIKON University General Hospital, a tertiary referral center with dedicated services in musculoskeletal oncology and complex primary and revision arthroplasty. The department serves as a national referral unit for tumor-related megaprosthetic reconstruction, while also managing a high-volume arthroplasty practice.

Participants were identified prospectively at the time of clinical evaluation for suspected periprosthetic joint infection (PJI). Enrollment occurred through two complementary pathways: patients either presented directly with symptoms or signs suggestive of PJI or were referred to our department by treating surgeons for further assessment based on clinical concern.

This dual approach ensured that all consecutive cases undergoing diagnostic synovial aspiration for possible infection at the index joint were captured during the study period. No patients were enrolled retrospectively, and no screening based on laboratory results or imaging alone was performed outside of clinically driven suspicion.

The index test under evaluation was synovial calprotectin, measured using a commercially available lateral-flow immunoassay (Lyfstone AS, Lysaker, Norway). The assay provides a quantitative calprotectin concentration (mg/L) based on the intensity of an antibody-mediated test line on the cassette. Synovial fluid (20–50 µL), obtained through routine diagnostic aspiration, was applied undiluted directly onto the test cassette according to manufacturer instructions. The assay was performed in the hospital clinical laboratory within one hour of aspiration.

All tests were conducted by orthopedic surgeons trained in the procedure by certified representatives of the manufacturer. Reaction time and visual interpretation followed the manufacturer’s protocol. The test was interpreted visually, and the resulting numerical calprotectin value was recorded. Test operators were blinded to the final infection reference standard and to all subsequent microbiological, operative, or adjudicated diagnostic information at the time of testing.

A predefined binary threshold of ≥50 mg/L was used to classify a test as positive, while values < 50 mg/L were considered negative. Although the manufacturer recommends a three-tier interpretation (negative, intermediate, positive), a binary threshold of ≥50 mg/L was selected based on prior literature. No indeterminate category was used, and no test failures or invalid results occurred.

Exploratory post hoc threshold observations were assessed separately and are reported descriptively in Results section.

The primary reference standard was a composite clinical–microbiological adjudication rather than the 2018 ICM criteria alone, as ICM classification was also assessed separately as a comparator endpoint. Because all patients underwent revision surgery, adjudication incorporated intraoperative tissue cultures, operative findings, and follow-up. Synovial calprotectin results were not available to clinicians establishing infection status, and the index test did not influence the diagnostic adjudication process. Patients were classified as infected when one or more major criteria were present, including two or more intraoperative tissue cultures yielding the same organism or the presence of a sinus tract communicating with the prosthesis. Clinical, serological, and operative findings, including pain, erythema, swelling, drainage, abnormal serum or synovial inflammatory markers, or purulent-appearing tissue, were not considered sufficient as standalone indicators, but contributed to the diagnostic decision only when interpreted in combination with microbiological results or major clinical criteria. A minimum one-year clinical follow-up was performed for all patients; this follow-up did not form part of the primary diagnostic classification but served to validate the initial determination, particularly in cases with limited or inconclusive intraoperative microbiological data. Patients were classified as not infected when intraoperative cultures were negative, major diagnostic criteria were absent, and no clinical or radiological evidence of infection was observed during the one-year surveillance period.

The clinicians responsible for establishing the reference standard diagnosis were blinded to the synovial calprotectin results, which were not available to them at the time of adjudication. Final infection classification was based solely on clinical, laboratory, operative, and microbiological information. Thus, the index test and reference standard were interpreted independently, preventing diagnostic review bias.

Clinical, laboratory, and intraoperative variables were collected prospectively at the time of evaluation for suspected periprosthetic joint infection (PJI) and during the subsequent revision surgery. Demographic data (age, sex), implant characteristics (joint involved, megaprosthesis vs. primary vs. revision arthroplasty), and clinical presentation (pain, swelling, erythema, drainage, fever) were recorded. Serum inflammatory markers, including C-reactive protein (CRP) and erythrocyte sedimentation rate (ESR), were obtained as part of routine diagnostic workup. Synovial fluid analysis included leukocyte count, polymorphonuclear percentage (PMN%), Gram stain, and culture. Synovial α-defensin testing and leukocyte esterase strip testing were not routinely available within our institutional diagnostic pathway during the study period and were therefore not collected.

Pre-aspiration antibiotic exposure, defined as systemic antibiotic administration within the 2 weeks preceding diagnostic aspiration, time from the most recent surgery on the index joint to biomarker sampling, and PJI timing category, defined as early (<3 months), delayed (3 months to <2 years), or late (≥2 years) infection, were documented for all infected cases. Intraoperative findings at revision surgery, including the presence of purulence, intraoperative pathology, and the number and results of tissue cultures, were recorded for every patient.

A broad set of variables was collected for descriptive and diagnostic accuracy analyses. For exploratory comparative analyses between megaprosthesis and non-megaprosthesis patients, a restricted set of prespecified covariates, specifically joint type, CRP category (<10 vs. ≥10 mg/L), infection timing category, pre-aspiration antibiotic exposure, and age, was used for matching and for constructing propensity scores in the sensitivity analysis. Matching was exact for the categorical covariates, while age was matched within a ±15-year window. All data were prospectively entered into a standardized electronic database immediately after clinical assessment and updated following revision surgery and microbiology reporting.

Diagnostic accuracy metrics for synovial calprotectin were calculated using the infection reference standard as the comparator. For the primary analysis in the megaprosthesis cohort, true positives (TP), false positives (FP), true negatives (TN), and false negatives (FN) were tabulated, and sensitivity, specificity, positive predictive value (PPV), negative predictive value (NPV), positive likelihood ratio (LR+), and negative likelihood ratio (LR−) were computed accordingly. Ninety-five percent confidence intervals (95% CIs) for all binomial accuracy metrics were calculated using the Wilson method, which provides stable interval estimates when proportions approach 0 or 1. Likelihood ratio confidence intervals were estimated on the log scale when applicable. The diagnostic performance of continuous calprotectin values was further assessed by calculating the area under the receiver operating characteristic (ROC) curve (AUC). A 95% confidence interval for the AUC was also calculated.

To contextualize calprotectin performance, a low-CRP subgroup analysis (CRP < 10 mg/L) was performed within the megaprosthesis cohort. Agreement between calprotectin classification (≥50 mg/L) and the final infection category assigned according to the 2018 International Consensus Meeting (ICM) criteria, used in this study solely for comparative purposes, was evaluated using Cohen’s κ, and directional disagreement was assessed using McNemar’s exact test.

Comparative analyses between megaprosthesis and non-megaprosthesis patients were conducted as exploratory contextual analyses. Among non-megaprosthesis patients with definitive infection reference classification, 23 were available prior to matching. The primary comparative method consisted of a 1:1 matched analysis using exact matching on joint type, CRP category, infection timing, and pre-aspiration antibiotic exposure, together with age matching within ±15 years. Nine megaprosthesis cases were successfully matched to nine non-megaprosthesis controls. The remaining 14 non-megaprosthesis patients were unmatched because no counterpart satisfied all prespecified matching criteria. A propensity score–matched sensitivity analysis was performed to assess whether the direction of the comparative findings remained consistent under an alternative matching strategy. Propensity scores were generated using logistic regression, including the same covariates used for the primary matched analysis, and nearest-neighbor matching without replacement was applied using a 0.2 caliper. Because of the limited sample size, both comparative analyses were interpreted descriptively and were not intended for formal between-group inference.

Statistical analyses were performed using standard methods for diagnostic accuracy studies, and no imputation was performed because no missing data were present for variables used in diagnostic calculations.

Revision surgery with intraoperative tissue sampling, constituting the reference standard, was performed within one month of synovial calprotectin testing in all patients, corresponding to the period required to complete the diagnostic evaluation for suspected periprosthetic joint infection (PJI). Treating surgeons responsible for establishing the reference standard diagnosis had access to all relevant clinical, laboratory, and operative information but were blinded to calprotectin results, ensuring that the index test and reference standard were interpreted independently.

The study was conducted in accordance with the Declaration of Helsinki and approved by the Institutional Review Board of the National and Kapodistrian University of Athens, First Department of Orthopaedics, ATTIKON University General Hospital (protocol code 508/06-07-2023).

## 3. Results

During the study period, 20 consecutive patients with tumor-related megaprosthetic reconstructions underwent diagnostic evaluation for suspected periprosthetic joint infection (PJI) and were prospectively enrolled. All patients had prior arthroplasty of the index joint, underwent synovial fluid aspiration with calprotectin testing, and subsequently proceeded to revision surgery in which intraoperative tissue cultures were obtained. Each patient completed a minimum of one year of clinical follow-up, allowing confirmation of infection status in all cases. No patients were excluded due to insufficient synovial sample, assay failure, or incomplete diagnostic information.

According to the predefined composite reference standard, 13 of 20 patients (65%) were classified as infected. Baseline demographic, clinical, and laboratory characteristics of the megaprosthesis cohort, stratified by infection status, are summarized in Table 1.

No patient in the cohort had a history of radiotherapy. Soft-tissue flap coverage was used selectively in proximal tibia megaprosthesis reconstructions, while prior chemotherapy exposure was present in a subset of patients but was heterogeneous in timing relative to biomarker sampling.

Using a predefined threshold of ≥50 mg/L, synovial calprotectin demonstrated high diagnostic performance for detecting periprosthetic joint infection in the megaprosthesis cohort. Of the 20 patients evaluated, the confusion matrix revealed 12 true positives, 7 true negatives, no false positives, and one false negative. This corresponded to a sensitivity of 92.3% (95% CI 66.7–98.6) and a specificity of 100% (95% CI 64.6–100). The positive predictive value was 100% (95% CI 75.7–100), while the negative predictive value was 87.5% (95% CI 52.9–97.8). Calprotectin values were substantially higher in infected than in non-infected patients, with a median (IQR) of 186.5 mg/L (68.0–222.7) versus 13.0 mg/L (13.0–20.0), respectively. The corresponding ranges were 39.0–301.0 mg/L for infected patients and 13.0–35.0 mg/L for non-infected patients.

**Figure 1:**
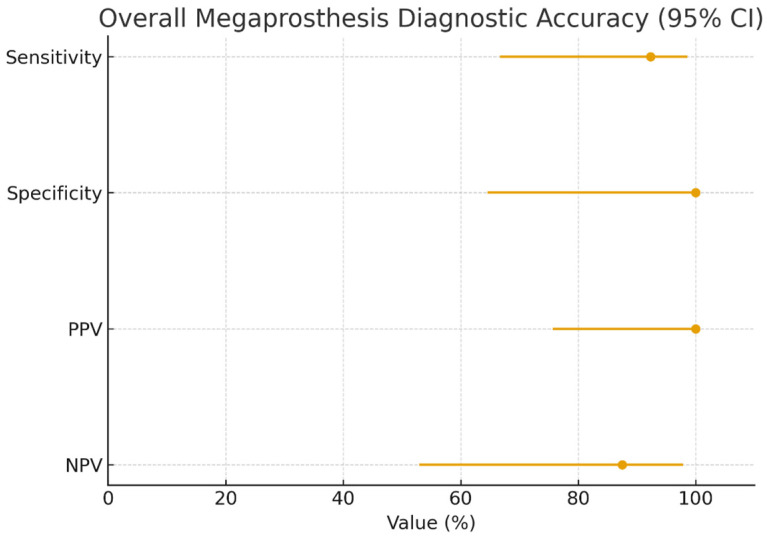
Forest plot of overall diagnostic accuracy for calprotectin in megaprosthesis patients, with 95% confidence intervals. Point estimates and Wilson 95% confidence intervals are shown for sensitivity, specificity, PPV, and NPV in the full megaprosthesis cohort (n = 20). Diagnostic accuracy was calculated against the infection reference standard.

The absence of false-positive results resulted in an infinite positive likelihood ratio (LR+), indicating strong rule-in capability, whereas the negative likelihood ratio (LR−) was 0.08 (95% CI 0.01–0.51), consistent with strong rule-out performance. When analyzed as a continuous variable, calprotectin showed complete separation between infected and non-infected cases in this dataset, with an area under the ROC curve (AUC) of 1.00 (95% CI, 1.00–1.00). Given the modest sample size, this finding should be interpreted cautiously. These findings are summarized in Table 2 and depicted in Figure 1.

A subgroup of 10 megaprosthesis patients presented with low systemic inflammation, defined as CRP < 10 mg/L. Within this subgroup, four patients were classified as infected according to the reference standard. Using the ≥50 mg/L threshold, synovial calprotectin correctly identified three of four infections (TP = 3) and correctly classified all non-infected cases (TN = 6, FP = 0), with one false-negative result. This corresponded to a sensitivity of 75%, specificity of 100%, PPV of 100%, and NPV of 85.7%, with an infinite positive likelihood ratio and a negative likelihood ratio of 0.25. These results indicate that calprotectin retains strong diagnostic performance even in megaprosthesis patients with low CRP values, where standard serum inflammatory markers are least informative.

In an exploratory descriptive subgroup analysis, synovial calprotectin demonstrated a sensitivity of 85.7% and specificity of 100% in antibiotic-exposed patients, compared with 100% sensitivity and 100% specificity in unexposed patients. Among infected patients, median CRP and calprotectin values were higher in the antibiotic-exposed subgroup than in the unexposed subgroup, with median CRP values of 43.0 mg/L versus 14.3 mg/L and median calprotectin values of 186.5 mg/L versus 165.0 mg/L, respectively. Given the small subgroup sizes and the non-stratified nature of the cohort, these findings were interpreted descriptively without formal between-group hypothesis testing.

Agreement between calprotectin (≥50 mg/L threshold) and the 2018 International Consensus Meeting (ICM) infection classification was assessed in the 17 megaprosthesis patients with definitive ICM categories (infected or aseptic); three cases categorized as “inconclusive” by the ICM criteria were excluded from agreement analysis. Calprotectin demonstrated substantial agreement with the ICM classification, with a Cohen’s κ of 0.76, indicating a high level of concordance between the two diagnostic approaches. The pattern of discordance was balanced, yielding a McNemar exact *p*-value of 1.00, which indicates no directional disagreement between calprotectin and the ICM-based classification.

Three megaprosthesis patients were classified as “inconclusive” according to the 2018 ICM criteria. Among these, two were ultimately determined to be infected by the composite reference standard, and one was not infected. Using the prespecified calprotectin threshold (≥50 mg/L), the test correctly identified two of the three inconclusive cases, one true infection and one true aseptic case, yielding a 66.7% correct reclassification rate within this diagnostically challenging subgroup.

The single misclassification corresponded to an infected case with a calprotectin value of 39 mg/L, which was the lowest value observed among infected patients in this dataset. This post hoc observation suggests that a lower threshold would have reclassified that case correctly; however, it is exploratory, data-derived, and should not be interpreted as a validated diagnostic cutoff.

**Figure 2:**
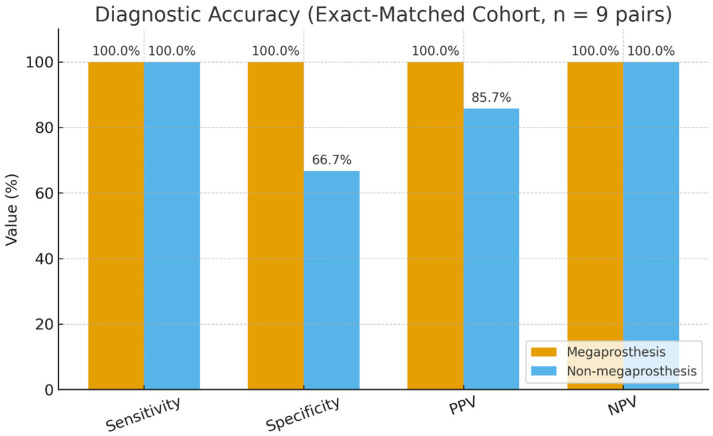
Diagnostic accuracy of synovial calprotectin in exact-matched megaprosthesis and non-megaprosthesis cohorts (n = 9 pairs). Bar chart comparing sensitivity, specificity, PPV, and NPV for calprotectin (≥50 mg/L) between megaprosthesis patients and matched non-megaprosthesis controls. Matching was exact for joint type, CRP category, infection timing, and antibiotic exposure, with age matched within ±15 years.

In the matched cohort, calprotectin correctly classified all megaprosthesis patients (sensitivity 100% and specificity 100%). In contrast, a single false-positive result was observed among the matched non-megaprosthesis controls, yielding 100% sensitivity and 66.7% specificity in that group. These comparative findings are illustrated in Figure 2, which highlights consistently high sensitivity across both cohorts and a notably higher specificity in megaprosthesis patients.

A propensity score–matched sensitivity analysis was also performed to verify the consistency of the comparative results. Calprotectin performance remained closely aligned with that observed in the exact-matched cohort. In megaprosthesis patients, sensitivity was 90.9% and specificity 100%, while in matched non-megaprosthesis controls, sensitivity remained 100% and specificity 83.3% due to a single false-positive result. Although exploratory and not powered for hypothesis testing, the PSM findings mirrored the direction and magnitude of the primary matched analysis, supporting the robustness of the comparative findings in an exploratory descriptive sense.

These exploratory matched analyses suggest that the diagnostic performance of calprotectin in the megaprosthesis cohort was broadly similar to that observed in matched non-megaprosthesis controls. Given the small matched sample size, these comparisons should be interpreted descriptively and cautiously.

Among the 13 infected megaprosthesis cases, coagulase-negative staphylococci (CoNS) represented the most common pathogens, accounting for 61.5% of infections. The most frequently isolated species were *Staphylococcus epidermidis* (3 cases), *S. haemolyticus* (3 cases), and *S. hominis* (2 cases). *Staphylococcus aureus* was identified in 3 cases (23.1%), while Gram-negative organisms, including *Pseudomonas oryzihabitans*, *Pseudomonas aeruginosa*, *Escherichia coli*, *Klebsiella pneumoniae*, *Proteus mirabilis*, and *Achromobacter insolitus*, were each identified in one case (7.7%). A single fungal infection with *Aspergillus niger* was also detected. Polymicrobial infection occurred in 4 of 13 cases (30.8%). A detailed breakdown of microbial isolates is presented in Table 3.

## 4. Discussion

This prospective study suggests that synovial calprotectin may be a useful biomarker for diagnosing periprosthetic joint infection in patients with megaprostheses, showing high apparent diagnostic accuracy in this exploratory cohort. Calprotectin values were clearly higher in infected than in non-infected cases, and the assay retained useful performance in patients with low CRP. Agreement with the 2018 ICM classification was substantial, and calprotectin correctly reclassified two of three ICM-inconclusive cases. These findings support the potential value of calprotectin as an adjunctive rule-out biomarker in a clinically difficult setting characterized by substantial diagnostic uncertainty and high-morbidity revision surgery.

To our knowledge, this is the first prospective study assessing synovial calprotectin specifically in tumor megaprostheses. The inclusion of a matched non-megaprosthesis comparator cohort provided useful clinical context and suggested that calprotectin performance remained in the same range as that observed in more conventional arthroplasty patients. Rather than establishing superiority or equivalence, these exploratory comparative analyses support the view that the diagnostic utility of calprotectin is preserved even in the biologically and surgically complex setting of tumor megaprosthetic reconstruction.

Several recent meta-analyses have consistently demonstrated that synovial calprotectin is a reliable and accurate biomarker for the diagnosis of periprosthetic joint infection (PJI). Pooled data indicate high sensitivity (ranging from 92 to 94%) and specificity (approximately 93%), with area under the curve (AUC) values consistently above 0.93, supporting both diagnostic precision and clinical utility [18,19,23]. Notably, calprotectin performs particularly well as a rule-out test, with negative likelihood ratios frequently below 0.1, reflecting a strong ability to exclude infection in ambiguous cases, a pattern broadly consistent with our megaprosthesis cohort. This rule-out strength, supported by high negative predictive values across studies, reinforces its potential role in guiding high-stakes revision decisions in complex reconstructions [21].

Calprotectin can be measured via laboratory-based ELISA or lateral flow test (LFT), the latter used in our study. Recent studies show both methods provide high diagnostic accuracy, with ELISA yielding slightly higher values but no clinically meaningful difference [17,18]. Direct comparisons have shown that LFT performs comparably to ELISA while offering faster, point-of-care results, supporting its use in real-time clinical decision-making [24].

Recent data suggest that synovial calprotectin may perform less reliably in cases of implant loosening and osteolysis. Lazic et al. (2023) reported reduced accuracy and more false positives in patients with radiographic bone loss, likely due to non-infectious inflammatory responses [25]. Despite the pathophysiologic overlap with megaprosthesis cases, we did not observe this limitation in our cohort, where calprotectin maintained high diagnostic performance even in complex reconstructions.

Several high-quality reviews and meta-analyses have confirmed that synovial calprotectin has been shown to perform competitively with serum and synovial biomarkers like α-defensin, IL-6 and PCR [24,26,27]. However, α-defensin and leukocyte esterase were not routinely available in our institutional diagnostic pathway during the study period, precluding direct head-to-head comparison in this cohort. Accordingly, the present study should be interpreted as evaluating calprotectin as a practical adjunctive biomarker in a real-world megaprosthesis setting rather than as establishing superiority over other synovial tests. In our low-CRP subgroup, calprotectin remained informative when conventional serum markers were less helpful to detect infection in low-inflammatory or immunosuppressed presentations.

Our findings demonstrated strong concordance between synovial calprotectin and the 2018 ICM criteria, which remains the most widely accepted diagnostic framework for PJI and has served as the reference standard in many studies evaluating calprotectin in standard arthroplasty settings [11,20,24,28,29]. This alignment supports the biomarker’s diagnostic validity within a broader consensus-driven algorithm that incorporates both clinical and laboratory-based criteria. Notably, calprotectin also proved effective in clarifying inconclusive cases.

Although a threshold of ≥50 mg/L for synovial calprotectin has been widely adopted, originally proposed by Wouthuyzen-Bakker et al. (2018) and supported across multiple studies, there remains considerable variation in the literature regarding optimal cutoffs [17,21]. Some studies have suggested that patients with inflammatory arthritis or advanced implant loosening may require higher diagnostic thresholds due to underlying synovial inflammation that elevates calprotectin levels in the absence of infection [25,30]. In our exploratory analysis, however, a lower cutoff near 39 mg/L appeared to improve diagnostic sensitivity without compromising specificity, suggesting that megaprosthesis patients may not fall into those higher-threshold categories. This post hoc observation is best viewed as hypothesis-generating: it suggests that threshold optimization in megaprosthesis patients may be worthwhile and provides a concrete value to be tested in future validation cohorts, rather than a cutoff for immediate clinical adoption.

This is the first prospective study evaluating the diagnostic efficacy of synovial calprotectin in patients with megaprosthetic reconstruction after oncological resection. The inclusion of a matched arthroplasty control cohort strengthens internal validity by contextualizing performance against a well-characterized comparator group. Diagnostic behavior was assessed using multiple complementary metrics, and the use of both exact matching and propensity score matching further supports the robustness of the findings. Finally, the use of a simple lateral-flow assay supports the reproducibility of the testing methodology across institutions. Several limitations should be acknowledged. The modest sample size and revision-only, high pre-test probability design may have inflated the apparent diagnostic performance. The cohort was biologically heterogeneous, and variables such as perioperative adjuvants were not sufficiently structured for meaningful modifier analysis. In addition, the matched comparison analyses were exploratory and underpowered, comparator biomarkers were unavailable, and the post hoc threshold observation requires external validation.

## 5. Conclusions

Synovial calprotectin showed promising diagnostic performance for identifying periprosthetic joint infection in patients with megaprosthetic reconstructions, including in cases with low systemic inflammatory markers. Its substantial agreement with the 2018 ICM classification and its ability to clarify most ICM-inconclusive cases support its value as a rapid and accessible adjunctive biomarker in this diagnostically challenging setting. Larger multicenter studies are needed to validate these findings and to clarify optimal diagnostic thresholds in megaprosthesis patients.

## Figures and Tables

**Table 1:** Baseline characteristics and laboratory values of the megaprosthesis cohort (n = 20), stratified by infection status. Continuous variables are presented as median (interquartile range). Categorical variables are reported as counts and percentages. * metallosis/wear percentages based on available cases only. CRP: C-reactive protein; ESR: erythrocyte sedimentation rate; WBC: white blood cell count; PMN: polymorphonuclear neutrophils.

Variable	Overall (n = 20)	Infected (n = 13)	Not Infected (n = 7)
Age (years)	57.0 (34.0–72.0)	60.0 (45.0–76.0)	45.0 (27.0–57.5)
Sex (male)	14 (70.0%)	10 (76.9%)	4 (57.1%)
Preoperative aspiration culture positive	8 (40.0%)	8 (61.5%)	0 (0.0%)
CRP (mg/L)	9.23 (5.50–44.75)	25.00 (8.46–50.00)	6.00 (3.50–7.00)
ESR (mm/hr)	33.5 (20.0–42.5)	35.0 (32.0–50.0)	18.0 (14.5–20.0)
Systemic WBC (×10^9^/L)	7.10 (6.00–8.74)	8.00 (6.00–9.60)	6.50 (6.00–7.10)
Synovial WBC (K/μL)	1.15 (0.37–5.93)	2.00 (0.40–6.00)	0.50 (0.38–1.43)
PMN (%)	64.0 (38.8–85.0)	78.0 (63.0–89.0)	35.0 (30.0–61.0)
Antibiotics prior to aspiration (≤14 days)	9 (45.0%)	7 (53.8%)	2 (28.6%)
Sinus tract present	1 (5.0%)	1 (7.7%)	0 (0.0%)
Time from last surgery to aspiration (years)	1.00 (0.46–2.00)	0.90 (0.33–1.50)	2.00 (1.00–3.50)
Index reconstruction site			
Knee	16 (80.0%)	10 (76.9%)	6 (85.7%)
Hip	3 (15.0%)	2 (15.4%)	1 (14.3%)
Humerus	1 (5.0%)	1 (7.7%)	0 (0.0%)
Surgical stage			
Primary	5 (25.0%)	4 (30.8%)	1 (14.3%)
Revision	15 (75.0%)	9 (69.2%)	6 (85.7%)
Metallosis/wear present *	6/14 (42.9%)	3/8 (37.5%)	3/6 (50.0%)

**Table 2:** Diagnostic accuracy of synovial calprotectin (≥50 mg/L) in the megaprosthesis cohort (n = 20). Values include sensitivity, specificity, positive predictive value (PPV), negative predictive value (NPV), positive and negative likelihood ratios (LR+ and LR−), and area under the receiver operating characteristic curve (AUC), each with 95% confidence intervals. Diagnostic performance is calculated using the infection reference standard.

Metric	Value (95% CI)
Sensitivity	92.3% (66.7–98.6)
Specificity	100% (64.6–100)
Positive Predictive Value (PPV)	100% (75.7–100)
Negative Predictive Value (NPV)	87.5% (52.9–97.8)
Positive Likelihood Ratio (LR+)	∞ (no false positives observed)
Negative Likelihood Ratio (LR−)	0.08 (0.01–0.51)
AUC (calprotectin, continuous)	1.00 (95% CI, 1.00–1.00)

**Table 3:** Microbiological profile of infected megaprosthesis cases (n = 13). Shown are categories of microorganisms identified from preoperative aspiration and/or intraoperative cultures, with subgroup species and proportions among infected cases. CoNS: coagulase-negative staphylococci.

Microorganism Category	Subgroup	n (%)
Coagulase-negative staphylococci (CoNS)	*S. epidermidis*	3 (23.1%)
Coagulase-negative staphylococci (CoNS)	*S. haemolyticus*	3 (23.1%)
Coagulase-negative staphylococci (CoNS)	*S. hominis*	2 (15.4%)
Staphylococcus aureus	*S. aureus*	3 (23.1%)
Gram-negative organisms	*Pseudomonas oryzihabitans*	1 (7.7%)
Gram-negative organisms	*Pseudomonas aeruginosa*	1 (7.7%)
Gram-negative organisms	*E. coli*	1 (7.7%)
Gram-negative organisms	*Klebsiella pneumoniae*	1 (7.7%)
Gram-negative organisms	*Proteus mirabilis*	1 (7.7%)
Gram-negative organisms	*Achromobacter insolitus*	1 (7.7%)
Streptococci	*Streptococcus* spp.	1 (7.7%)
Fungi	*Aspergillus niger*	1 (7.7%)
Polymicrobial infections	-	4 (30.8%)

## Data Availability

The data presented in this study are available on reasonable request from the corresponding author. The data are not publicly available due to privacy and ethical restrictions, as they contain potentially identifiable clinical information from a small single-center cohort.
